# Median eminence blood flow influences food intake by regulating ghrelin access to the metabolic brain

**DOI:** 10.1172/jci.insight.165763

**Published:** 2023-02-08

**Authors:** Nicola Romanò, Chrystel Lafont, Pauline Campos, Anne Guillou, Tatiana Fiordelisio, David J. Hodson, Patrice Mollard, Marie Schaeffer

**Affiliations:** 1 Institute of Functional Genomics and; 2 BioCampus Montpellier, University of Montpellier, CNRS, INSERM, F-34094 Montpellier, France.; 3 Laboratorio de Neuroendocrinología Comparada, Laboratorio Nacional de Soluciones Biomiméticas para Diagnóstico y Terapia LaNSBioDyT, Science Facility, Universidad Nacional Autónoma de México, Mexico City, Mexico.; 4 Oxford Centre for Diabetes, Endocrinology and Metabolism (OCDEM), NIHR Oxford Biomedical Research Centre, Churchill Hospital, Radcliffe Department of Medicine, University of Oxford, Oxford, United Kingdom.; 5 Centre de Biologie Structurale, CNRS UMR 5048, INSERM U1054, University of Montpellier, Montpellier, France.

**Keywords:** Metabolism, Pericytes

## Abstract

Central integration of peripheral appetite-regulating signals ensures maintenance of energy homeostasis. Thus, plasticity of circulating molecule access to neuronal circuits involved in feeding behavior plays a key role in the adaptive response to metabolic changes. However, the mechanisms involved remain poorly understood despite their relevance for therapeutic development. Here, we investigated the role of median eminence mural cells, including smooth muscle cells and pericytes, in modulating gut hormone effects on orexigenic/anorexigenic circuits. We found that conditional activation of median eminence vascular cells impinged on local blood flow velocity and altered ghrelin-stimulated food intake by delaying ghrelin access to target neurons. Thus, activation of median eminence vascular cells modulates food intake in response to peripheral ghrelin by reducing local blood flow velocity and access to the metabolic brain.

## Graphical abstract

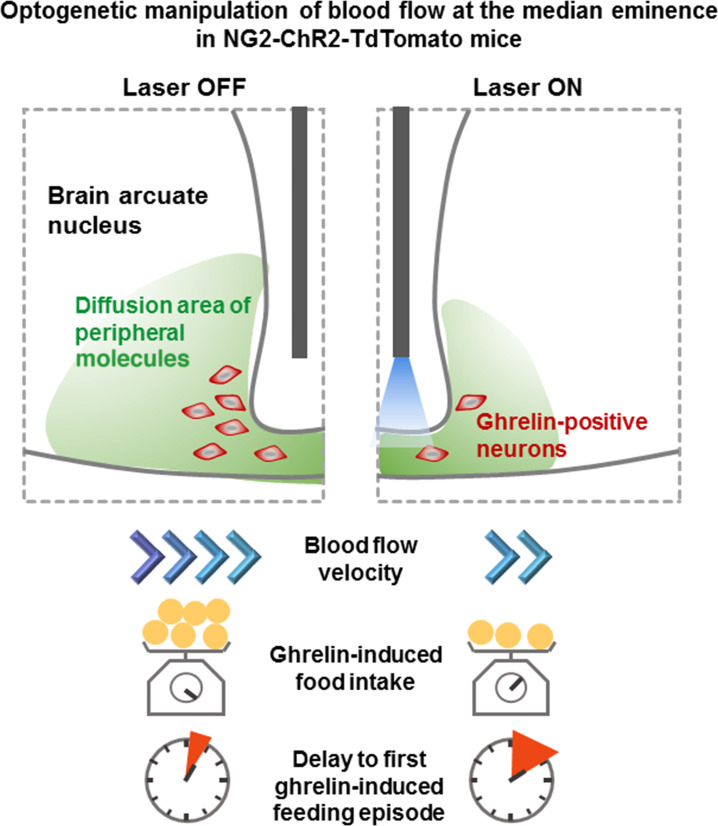

## Introduction

Adaptation of food intake to energy requirements is dependent on the ability of different brain centers to rapidly sense and integrate changes in blood levels of peripheral molecules. Although the regulation of communications between peripheral circulation and central integration compartments may play an integral part in controlling feeding behavior and energy balance (1), the mechanisms involved in this process remain poorly understood.

Many appetite-regulating peptides exclusively produced in the periphery are able to act on specific target sites in the brain to regulate food intake (2). The hypothalamic arcuate nucleus (ARH) constitutes the primary integration center (3, 4), where circulating factors act on functionally opposing neuronal populations to coordinate homeostatic responses and metabolism (4, 5). Thus, both access rate and distribution patterns of peripheral gut hormones into the ARH constitute key regulators of food intake. Although neurons are usually protected by the blood-brain barrier (BBB), the ARH is located in close proximity to the capillary plexus of the median eminence (ME), a key circumventricular organ (CVO). While ME tanycytes can actively transport blood-borne signals to regulate food intake at relatively slow kinetics (6), nutrient-dependent regulation of fast molecule access to the metabolic brain implicates fenestrated endothelial capillary loops (7–9). ME capillaries allow passive extravasation into the ARH of small circulating hormones (≤40 kDa in size) (7) and undergo profound remodeling in response to fasting-induced decrease in blood glucose in mice (8). Such remodeling involves vascular endothelial growth factor A–dependent (VEGFA-dependent) increases in ARH capillary fenestration and number of capillary loops, which promote rapid delivery of circulating appetite-regulating hormones (e.g., ghrelin) (8). Of note, VEGFA-dependent mechanisms are likewise involved in remodeling of the ME vasculature according to seasonal changes in energy requirements to aid central-peripheral communications (10). Thus, plasticity of hormone uptake mechanisms in the ARH plays a key role in the adaptive response to metabolic changes. As such, understanding the mechanisms that regulate molecule access to the key neuronal determinants of feeding behavior in the ARH, and potentially other CVOs, may be important for therapeutic development.

Among other mechanisms that influence access rate and distribution of peripheral gut hormones into the ARH, the role of capillary blood flow velocity at the level of the ME remains unclear. The ME microvasculature is composed of arterioles and capillaries, lined by contractile smooth muscle cells and pericytes, respectively (11). Pericytes have attracted increasing interest due to their role in regulating capillary blood flow (12–14), BBB integrity (15), and vascular permeability to circulating leptin (16). In addition, pericytes are implicated in the sustained blood flow decrease in the brain following ischemia, stroke (17), and development of pathologies such as cerebral arteriopathy (18, 19) and diabetic retinopathies (20). As such, we hypothesized that modulation of ME blood flow might be able to modulate food intake through regulation of peripheral molecules’ access to the ARH, thus providing an additional level of regulation in gut-brain crosstalk. Here, using selective optogenetic manipulation of ME mural cells and ghrelin injection as a food intake trigger in mice, we demonstrate that short-lived changes in ME blood flow are able to alter peptide hormone access to the ARH as well as food intake.

## Results

### Optogenetic activation of ME NG2-positive cells reduces local blood flow velocity through vessel constriction.

**Figure 1:**
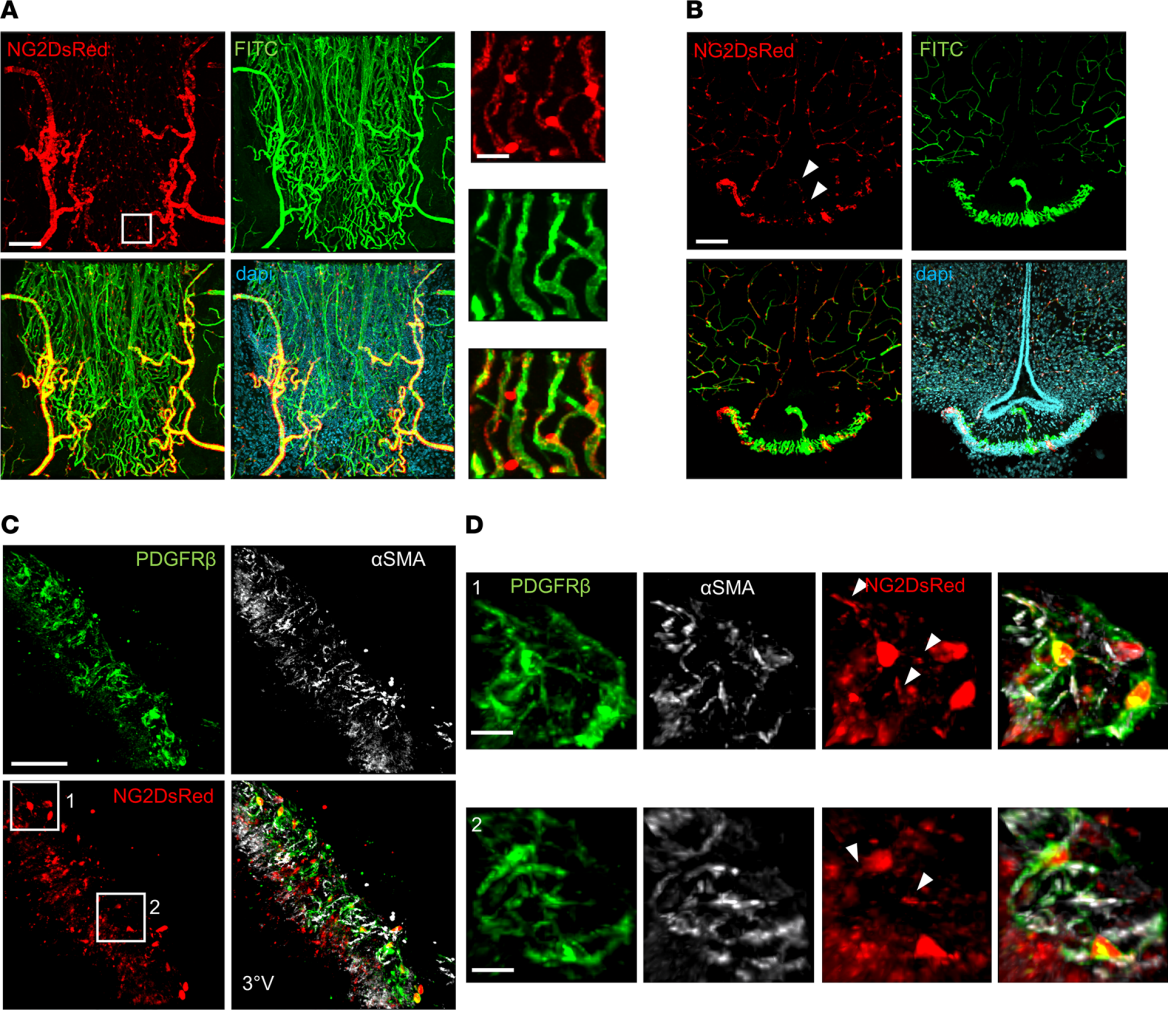
NG2-expressing cells line capillaries in the ME/ARH region. (**A**) Confocal images depicting a ventral view of the ME in an NG2DsRed mouse (left panel, scale: 100 μm, 50 μm *Z*-projection; red: NG2DsRed, green: FITC-lectin, blue: DAPI), showing NG2DsRed cellular bodies and processes line ME capillaries. Right panels (scale: 20 μm, 15 μm *Z*-projection) correspond to an enlargement of the boxed area on left. (**B**) Confocal images depicting a coronal view of the ME in an NG2DsRed mouse (left panel, scale: 100 μm, 200 μm *Z*-projection; red: NG2DsRed, green: FITC-lectin, blue: DAPI). Arrows indicate the position of NG2DsRed cells along capillary loop projections within the ME. (**C**) Confocal images depicting a coronal view of the ME in an NG2DsRed mouse (scale: 50 μm, 20 μm *Z*-projection; red: NG2DsRed, green: PDGFR-β, white: α-SMA; 3°V: third ventricle). (**D**) Enlargements of boxed areas in **C** (scale: 10 μm, 15 μm *Z*-projection; red: NG2DsRed, green: PDGFR-β, white: α-SMA). Arrows indicate NG2DsRed cell projections labeled with PDGFR-β and α-SMA.

**Figure 2:**
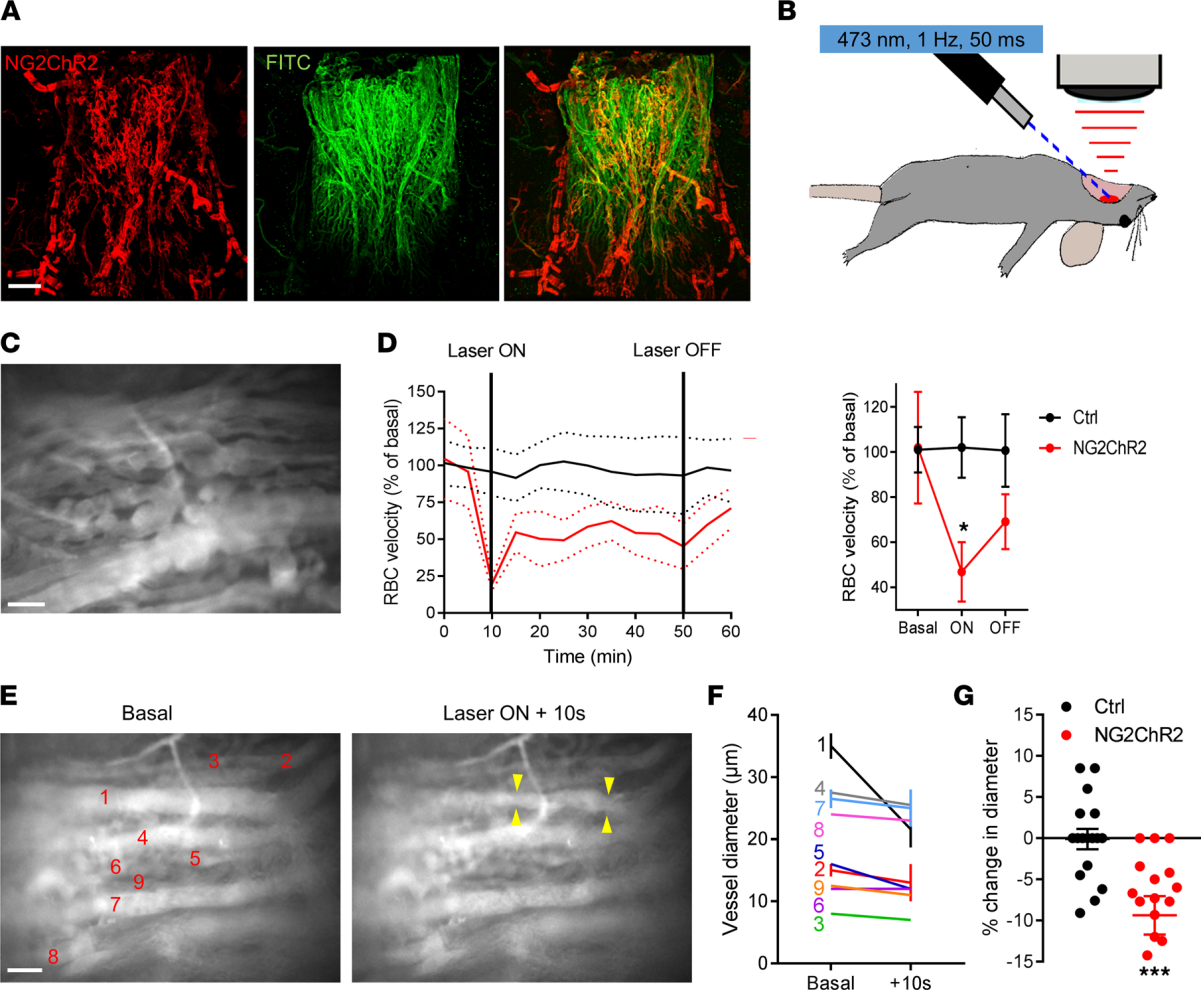
Optogenetic stimulation in NG2-ChR2 mice in vivo at the ME modifies blood flow and vessel diameter. (**A**) Confocal images depicting a ventral view of the ME in an NG2-ChR2 mouse (scale: 100 μm, 140 μm *Z*-projection; red: NG2-ChR2, green: FITC-lectin) and showing NG2DsRed cellular bodies and processes line ME capillaries. (**B**) Schematic of the in vivo optogenetic manipulation and imaging setup of blood flow at the ME (protocol adapted from ref. 45). Vessel parenchyma was labeled with D2-dextran. (**C**) Still image from an in vivo movie of blood flow at the ME in an NG2-ChR2 mouse at the start of laser stimulation (see also Supplemental Video 1) (scale: 50 μm, single *Z*-plane). (**D**) Quantification of RBC velocity in control and NG2-ChR2 mice before, during, and after 40 minutes of laser stimulation (473 nm, 50 ms, 1 Hz, 400 nm fiber at <0.5 mm of tissue). Data are presented as mean ± SEM. RBC velocity is significantly reduced during laser stimulation in NG2-ChR2 mice (*n* = 3–6 mice/condition, 5–10 vessels/mouse, *P < 0.05, 2-way ANOVA). (**E**) Still images from an in vivo movie of blood flow at the ME in an NG2-ChR2 mouse at the start of laser stimulation (left panel) and after 10 seconds of stimulation (right panel) (see also Supplemental Video 2) (scale: 50 μm, single *Z*-plane). Arrows indicate visible vessel constriction. Numbers indicate vessels analyzed in **F**. (**F**) Quantification of vessel constriction in movie in **E**. Data are presented as mean ± SEM. Diameter was measured along vessels 1 to 5 times in each vessel. Data are presented as mean ± SEM. (**G**) Quantification of percentage change in vessel diameters in the ME after more than 10 seconds of laser stimulation in control or NG2-ChR2 mice shows a reduction of mean vessel diameter in NG2-ChR2 mice during laser stimulation compared with basal conditions (*n* = 3 mice/condition, 5–10 vessels/mouse, ***P < 0.001, Mann-Whitney). Data are presented as mean ± SEM.

Neuroglial 2 (NG2) is a proteoglycan that is involved in cell adhesion, communication, and migration (21). NG2 is mainly expressed in perivascular cells in the ME but is essentially absent in endothelial cells (22, 23) (Supplemental Figure 1; supplemental material available online with this article; https://doi.org/10.1172/jci.insight.165763DS1). NG2-positive cell coverage at the level of the ME was assessed using mice expressing DsRed under the control of the NG2 promoter (NG2DsRed mice), which present homogeneous labeling of both cytoplasmic and cell protrusions in NG2-positive cells (12, 24, 25). NG2DsRed cells lined the dense capillary network of the ME (Figure 1A), as well as the fenestrated capillary loops projecting within the ME (Figure 1B) (7). NG2DsRed cells in the ME also expressed both the platelet-derived growth factor receptor–β (PDGFR-β), a marker commonly used to identify pericytes (15), and the contractile smooth muscle actin protein (α-SMA) (Figure 1, C and D). Of note, arteriolar smooth muscle cells also express α-SMA and NG2 (26–28). To allow precise functional interrogation of NG2-positive cell activity, the blue light–sensitive cation channel ChR2 was expressed in a Cre-dependent manner in NG2-Cre animals. As expected, a similar anatomical distribution of NG2-positive cells was seen at the ME level in NG2-Cre ROSA26-ChR2-tdTomato (NG2-ChR2) mice (Figure 2A). tdTomato labeling was, however, limited to the plasma membrane of NG2-positive cells in these mice. Blood flow was imaged after removal of the jaw bone in terminally anesthetized mice as reported (7), whereas laser stimulation was performed using optic fibers pointed at the level of the ME (Figure 2B). Vascular plasma was labeled using fluorescent dextran (Figure 2C) and blood flow recorded over 1 hour of laser stimulation in vivo. Optogenetic stimulation was performed using a 1 Hz stimulation with 50 ms pulses of 473 nm laser light. Light stimulation resulted in a prompt decrease in blood flow in NG2-ChR2 mice (Supplemental Video 1). After an initial large drop in flow velocity, blood flow showed a slight increase, without reaching the velocity observed under unstimulated conditions. In addition, the overall blood flow remained significantly reduced during 1 hour of laser stimulation (*P* < 0.05, 2-way ANOVA) (Figure 2D). Blood flow was not affected by laser stimulation in control littermates lacking ChR2 (Figure 2D). Notably, the effects of optogenetic stimulation were readily reversible, with red blood cell (RBC) velocity returning to baseline levels after switching off the laser (Figure 2D). The decrease in blood flow was associated with contraction of vessels in NG2-ChR2 mice (ranging from ~5% to 40% reduction in vessel diameter, mean ~10%; Figure 2, E–G, and Supplemental Video 2), with a stronger effect on larger vessels than small capillaries. Thus, conditional activation of ME NG2-positive cells impinges on local blood flow velocity, presumably through vessel constriction.

### Optogenetic activation of ME NG2-positive cells reduces and delays ghrelin-stimulated food intake.

**Figure 3:**
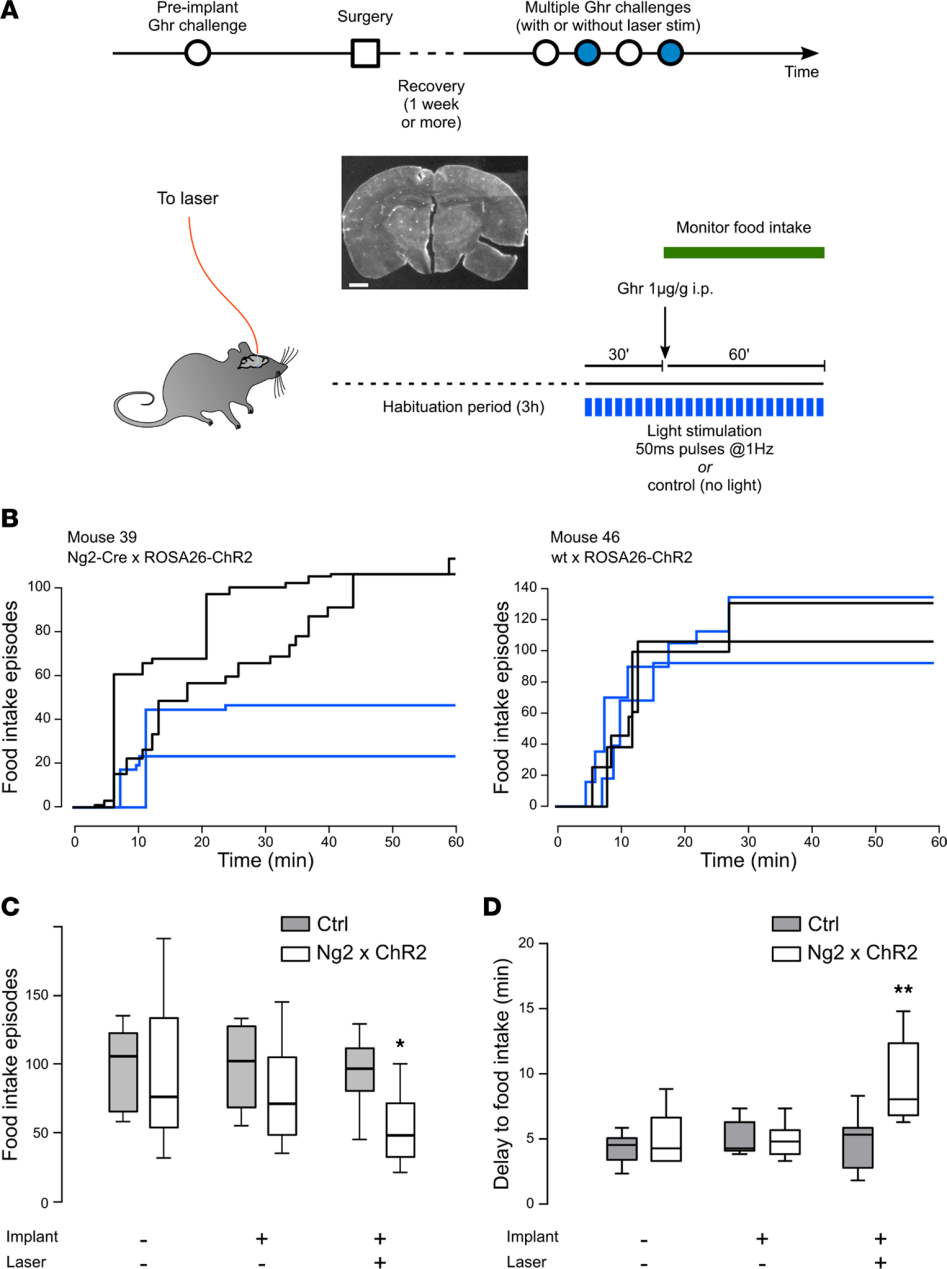
Optogenetic stimulation of NG2-positive cells at the ME decreases and delays ghrelin-stimulated food intake. (**A**) Schematic of experimental design. Mice were analyzed preimplantation, and several times postimplantation (with or without laser stimulation), leaving at least 1 week recovery postsurgery and between ghrelin (Ghr) challenges. After 3 hours’ habituation attached to the optic fiber, the laser was switched on, and ghrelin was injected i.p. 30 minutes after switching on the laser. Picture depicts a representative coronal slice of an implanted brain. Scale: 2 mm. (**B**) Food intake episodes over time after injection of ghrelin i.p. in a representative NG2-ChR2 mouse (left) and a single transgenic ROSA26-ChR2 mouse (right). Individual lines correspond to repetitions of the experiment in the same animal. Black and blue lines correspond to feeding episodes elicited by ghrelin injection when mice are attached to optic fibers and laser is off or on, respectively. (**C**) Total number of food intake episodes over the course of 1 hour after ghrelin injection i.p. (*n* = 6–7 mice/condition, *P < 0.05, mixed-effect model). (**D**) Delay to first feeding episode after ghrelin injection i.p. (*n* = 6–7 mice/condition, **P < 0.01, mixed-effect model). Boxes represent median with interquartile range; whiskers represent min to max range.

To determine whether activation of NG2-positive cells at the ME modulates peripheral hormone sensing in the metabolic brain, optical fibers were implanted above the ME in either NG2-ChR2 mice or control littermates for optogenetic stimulation in freely moving animals. Ghrelin-stimulated hourly food intake was measured multiple times in individual mice, before and after fiber implantation, with or without laser stimulation, in randomized order between different animals (Figure 3A). Ghrelin injection resulted in a rapid and significant increase in food intake in both NG2-ChR2 and control mice (Supplemental Figure 2A), and total food intake was proportional to the number of eating episodes (Supplemental Figure 2B). Laser stimulation reduced the total number of ghrelin-stimulated eating episodes in NG2-ChR2 mice but not in control animals (Figure 3, B and C, *P* < 0.05, mixed-effect model). Furthermore, laser stimulation induced a significant delay (~3 minutes) in the lag to first eating episode after ghrelin injection in NG2-ChR2 animals compared with control animals (Figure 3D, *P* < 0.01, mixed-effect model). In contrast, laser stimulation of NG2-ChR2 mice under basal conditions (i.e., without ghrelin stimulation) did not modify feeding behavior compared to control littermates (Supplemental Figure 3).

### Optogenetic activation of ME NG2-positive cells impinges on peripheral molecule access to the metabolic brain.

**Figure 4:**
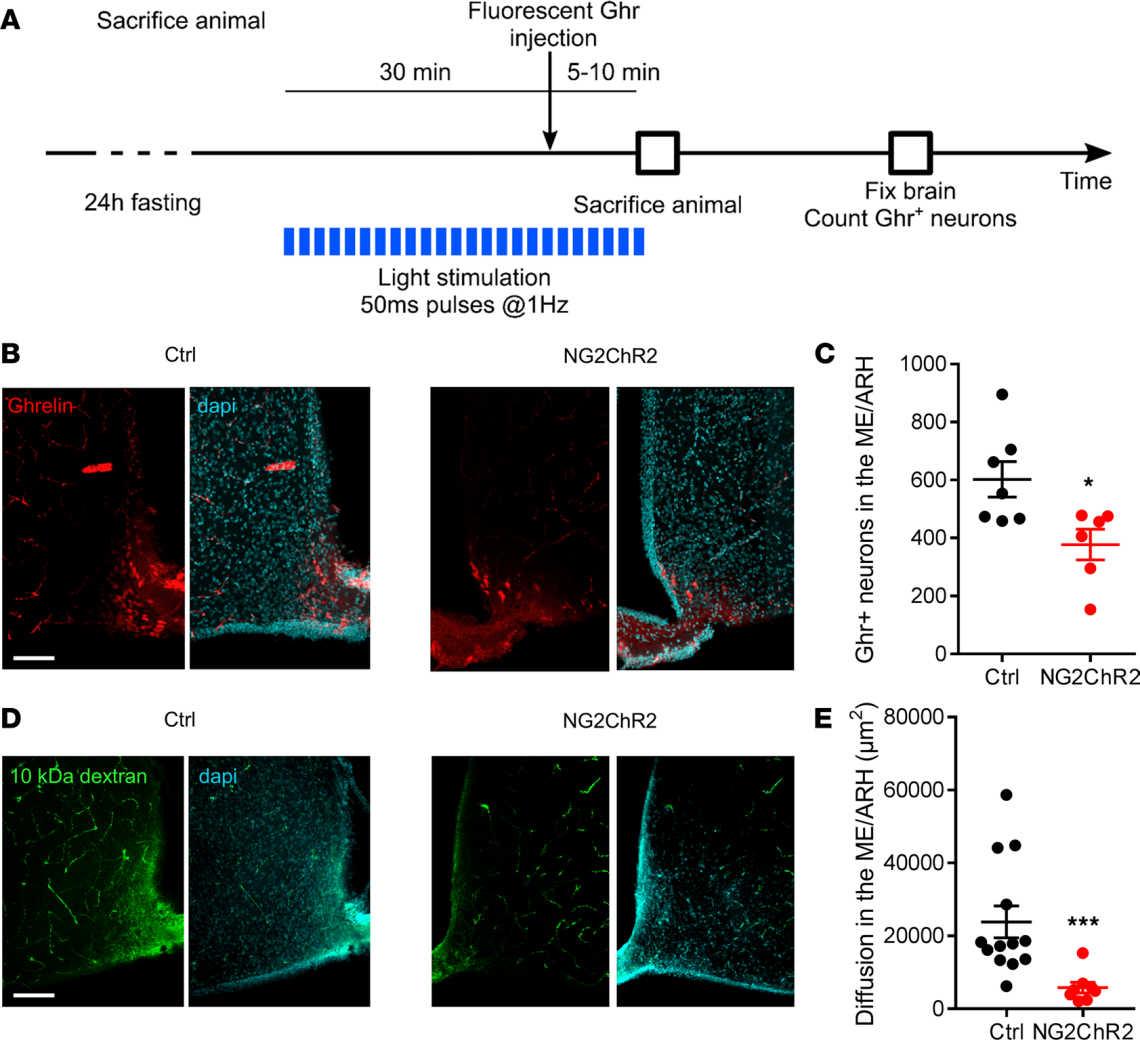
Optogenetic stimulation of NG2-positive cells at the ME decreases peripheral ghrelin access to the ME/ARH region. (**A**) Schematic of experimental design. Laser stimulation was applied to 24-hour-fasted animals for 30 minutes before injection of fluorescent ghrelin injection i.v. Sacrifice was 5–10 minutes postinjection. Ghr, ghrelin. (**B**) Confocal images of ME/ARH region 10 minutes following i.v. injection of fluorescent ghrelin (red) in a control mouse (left) and in an NG2-ChR2 mouse (right) subjected to 30 minutes of continuous laser stimulation at the level of the ME (473 nm, 50 ms, 1 Hz) (blue: DAPI) Scale: 100 μm, 12 μm *Z*-projection. (**C**) Quantification of ghrelin-labeled neurons in the whole ME/ARH region under optogenetic stimulation (15–24 slices/mouse, *n* = 6–7 mice/condition). Numbers were normalized to correspond to total ME length (1,200 μm; 24 slices, 50 μm thick). Data are presented as mean ± SEM. Optogenetic stimulation reduces the number of ghrelin-labeled neurons in the ME/ARH at 10 minutes after injection of ghrelin in NG2-ChR2 mice (*P < 0.05, Mann-Whitney test). (**D**) Confocal images of ME/ARH region 5–10 minutes following i.v. injection of fixable 10 kDa dextran-FITC (green) in a control mouse (left) and in an NG2-ChR2 mouse (right) subjected to 30 minutes of continuous laser stimulation at the level of the ME (473 nm, 50 ms, 1 Hz) (blue: DAPI). Scale: 100 μm, 16 μm *Z*-projection. (**E**) Quantification of the diffusion area of fixable 10 kDa dextran-FITC into the ME/ARH region under optogenetic stimulation (2–4 slices/mouse, *n* = 3 mice/condition). Data are presented as mean ± SEM. Optogenetic stimulation reduces the area of diffusion of 10 kDa dextran into the ME/ARH at 5–10 minutes postinjection in NG2-ChR2 mice (***P < 0.001, Mann-Whitney test).

To quantify the effects of ME NG2-positive cell activation on peripheral molecule access to the metabolic brain, first we used a fluorescent ghrelin analog that is internalized by target neurons in the ARH upon growth hormone secretagogue receptor (GHSR1a) binding (7). Fluorescent ghrelin was injected into the tail vein following 30 minutes of light stimulation (50 ms, 1 Hz pulses), before sacrificing animals and quantifying the total number of ghrelin-positive neurons in the ME/arcuate region (5–10 minutes postinjection) (Figure 4A). Light stimulation significantly reduced the number of neurons labeled with fluorescent ghrelin in NG2-ChR2 animals compared with their control littermates (~30% reduction; Figure 4, B and C; *P* < 0.05, Mann-Whitney test). Second, we assessed the diffusion of a fixable fluorescent inert sugar (10 kDa dextran-FITC) within the size range of most peptide hormones. Fluorescent dextran was injected into the tail vein following 30 minutes of light stimulation (50 ms, 1 Hz pulses), before sacrificing animals and quantifying molecule diffusion area in the ME/arcuate region (5–10 minutes postinjection). Light stimulation significantly reduced the diffusion area of fluorescent dextran in NG2-ChR2 animals compared with their control littermates (Figure 4, D and E, *P* < 0.001, Mann-Whitney test). Thus, activation of ME NG2-positive cells modulates food intake in response to peripheral ghrelin by reducing local blood flow velocity and access to target sites, rather than relying on an active uptake mechanism. More generally, activation of ME NG2-positive cells modulates access of circulating molecules to the ME/ARH.

To test effects of ME NG2-positive cell manipulation on neuron functional activation in the metabolic brain, we injected ghrelin following 30 minutes of light stimulation (50 ms, 1 Hz pulses), sacrificed animals 2 hours postinjection, and quantified c-Fos–positive nuclei in ME/ARH slices (Supplemental Figure 4). Light stimulation did not significantly modify the number of c-Fos–labeled nuclei in NG2-ChR2 animals compared to their control littermates (Supplemental Figure 4, 1-way ANOVA).

## Discussion

Rapid sensing and integration of changes in circulating appetite-modifying peptide levels by the metabolic brain ensure maintenance of energy homeostasis. Key neuronal determinants of feeding behavior in the ARH are strategically located in close apposition to the ME, a CVO, which dynamically regulates molecule access to the metabolic brain in a nutrient-dependent manner (29). Since mechanisms involved in peripheral hormone access to the ARH may be important for developing new antiobesity therapies, we sought to investigate the role of contractile vascular cells in modulating molecule access to the metabolic brain. To do so, we combined optogenetic manipulation of ME NG2-positive cells with ghrelin-stimulated food intake. NG2-positive cell activation in the ME induced a local reduction in blood flow velocity, decreased and delayed ghrelin-stimulated food intake, and delayed ghrelin access to the ARH. We thus demonstrate that activation of NG2-positive mural cells in the ME alters food intake in response to ghrelin injection in the periphery, through a reduction of blood flow velocity, which delays ghrelin access to target neurons in the ARH.

NG2 is expressed by different cell types in the ARH. First, pericytes wrap around nonfenestrated brain endothelial cells, contributing to the endothelial barrier properties of the central nervous system (CNS) (16, 30–32). CNS pericytes are a heterogeneous cell population (33), commonly identified by the NG2 and PDGFR-β markers (34). In addition, α-SMA labels contractile pericytes on capillaries (12, 27, 28). Second, NG2 is expressed in morphologically distinct arteriolar smooth muscle cells, also expressing α-SMA (14, 35). Finally, NG2 is expressed in oligodendrocyte precursor cells (OPCs), also called NG2-glia (36). Therefore, optogenetic stimulation of NG2-ChR2–expressing cells using a 200 μm diameter fiber optic aimed at the ventral ME likely targets OPCs, arteriolar α-SMA–positive cells, and pericytes and does not target endothelial cells, which do not express (or express very low levels of) NG2 (23).

It has been shown that pericytes are electrically responsive, enabling optogenetic control of their activity (14, 27, 28, 35). In accordance with a role for pericytes in blood flow regulation described previously in the cerebellum (13) and the cortex (12), manipulation of NG2-positive cell activity through optogenetic stimulation in vivo was able to modify blood flow in the ME. Very small (5%–10%) and sometimes unnoticeable variations in vessel diameter were able to lead to great changes in capillary blood flow (~50%). Since capillary diameters are usually similar to RBC width, minor vessel constrictions resulting from pericyte and/or arteriolar α-SMA–positive cell activity can lead to relatively large increases in friction and resistance to RBC flow. In addition, it has been suggested that contraction of pericyte longitudinal processes could alter capillary blood flow without changes in diameter, through stiffening of the vascular wall (27). It is, however, unclear how activation of OPCs might alter blood flow.

Optogenetic stimulation of ME NG2-positive cells delayed the time of the first ghrelin-stimulated eating episode by approximately 3 minutes, which was accompanied by an overall decrease in food intake over the 1-hour experiment time. This decrease in food intake could not solely be explained by the delay in ghrelin-stimulated feeding. Indeed, the 5% reduction in time during which mice ate over an hour (corresponding to the 3-minute delay) is unlikely to be entirely responsible for the 30% reduction in number of feeding episodes observed in laser-stimulated NG2-ChR2 compared with control mice. Although reduced blood flow in the ME may delay ghrelin access to the metabolic brain and thereby increase the lag to first eating episode, alteration of ghrelin delivery to the ARH may also lead to prolonged effects on GHSR1a signaling, altering overall food intake during the 1-hour period after ghrelin injection. Indeed, previous data on ghrelin-GHSR1a stimulation demonstrated that the most robust response was observed when the triggering stimulus was delivered as a bolus rather than an infusion (37, 38). Although 2 hours of ghrelin infusion was unable to induce a change in c-Fos activation in the present study, we note that longer time scales might be necessary to invoke ARH neuron activation (39), as well as allow for other slower receptor-mediated transport mechanisms or transcytosis mechanisms to be triggered (40). Further studies with high spatiotemporal resolution are required to analyze early changes in neuronal activation, e.g., fiber photometry or 2-photon imaging of calcium activity or voltage dynamics in defined ARH neuronal populations. Together, these observations suggest that vascular cells modulate the specific temporal pattern of ghrelin signaling in the ARH, through fine control of the concentration and length of exposure to the hormone, mediated by changes in blood flow. Although reduced blood flow might locally impact oxygen and nutrient delivery, gasses and small molecules are highly diffusible and rely less on vessel fenestration for diffusion (41). Consistent with this, laser stimulation of NG2-ChR2 mice under basal conditions (i.e., without ghrelin stimulation) did not modify feeding behavior compared to control littermates, suggesting neurons receive sufficient oxygen and nutrient supply through vessels unaffected by laser stimulation. Further (challenging) experiments measuring local in vivo oxygen tension would help to clarify this issue.

Ghrelin was used in the present study since it is an acute and rapid trigger of the feeding response in the fasted state (within minutes) (37). While it would be of interest to study the impact of ME blood flow on the entry of other hormones into the ARH, inhibition of food intake by leptin or glucagon-like peptide 1 receptor agonist injection, for instance, may require a much longer observation time and continuous laser stimulation, since feeding episodes occur mainly during the nocturnal phase and are relatively spread out. In addition, while fenestrated capillaries of ME provide a route of entry for leptin (8), effects on the ARH are largely mediated by leptin transported from the cerebrospinal fluid through tanycytes in a receptor-mediated process (42). Thus, coexistence of different transport mechanisms acting in conjunction and in relay may add to the complexity of analyzing leptin effects. Together with the recently described role for pericytes and OPCs in the modulation of leptin signaling in the hypothalamus (16, 36), our data support a central role for vascular mural cells, in addition to tanycytes, in modulating feeding behavior and possibly as a general gatekeeper to modulate hormone entrance to the brain.

Consistent with an effect of reduced blood flow in the ME on altered ghrelin access to the metabolic brain, and GHSR1a-ligand complex internalization by endocytosis in a time-dependent manner (38), fewer (~30%) ghrelin-positive neurons could be observed in the ME/arcuate region in laser-stimulated NG2-ChR2 mice compared with control mice 5–10 minutes postinjection of a bioactive fluorescent ghrelin. However, the location of labeled neurons was still restricted to ARH regions, where the main ghrelin-sensitive food intake–regulating neurons reside (4, 5), as previously shown (7). Reduced/delayed activation of ARH neurons may contribute to the lasting effects of laser stimulation on food intake over the 1-hour experiment since the number of ghrelin-positive neurons was still reduced in NG2-ChR2 compared with control mice 5–10 minutes following bioactive fluorescent ghrelin injection (i.e., a time point beyond the lag to first ghrelin-stimulated feeding episode). We cannot exclude that changes in vascular permeability contribute to the reduction in ghrelin-labeled cell number, since pericytes are able to modulate vessel permeability (16, 43). However, it is unlikely that changes in permeability would further affect extravasation since ghrelin already has a very high extravasation rate in the ME because of its low molecular weight (7). In addition, control of vessel permeability by pericytes has been described in vessels outside the ARH, and it is not clear whether modulation of pericyte activity could reduce permeability in fenestrated vessels. Supporting a direct role of pericytes in the control of molecule extravasation into the ME/ARH, the extent of diffusion of fixable 10 kDa dextran-FITC was reduced in laser-stimulated NG2-ChR2 mice versus control mice 5–10 minutes postinjection (7). However, further experiments are warranted to differentiate effects of reduced blood flow and vascular permeability on molecule diffusion.

In summary, we have shown that in vivo optogenetic activation of ME NG2-positive cells modulates ghrelin-stimulated food intake by reducing local blood flow velocity and ghrelin access to the target site, thus unveiling a new angle of metabolic regulation with important implications for therapeutic design. Future studies are warranted to assess ME vascular mural cells’ function in the context of metabolic diseases to evaluate their potential as a therapeutic target.

## Methods

### Mice.

C57BL/6 mice were purchased from Janvier-SAS. NG2DsRed, NG2-Cre, and ROSA26-ChR2 (H134R)-tdTomato mice (44) on a C57BL/6 background were sourced from The Jackson Laboratory. In all experiments, 8- to 15-week-old mice were used. ROSA26-ChR2-Tomato mice were crossed to NG2-Cre mice to allow specific expression of light-sensitive ion channels in NG2-expressing cells, a marker commonly used to identify pericytes (25), to impose a firing tempo by depolarization, opening of voltage-activated calcium channels, and pericyte contraction using laser flashes (473 nm) (12). Controls consisted of a combination of littermate WT, NG2-Cre, and ROSA26-ChR2-Tomato mice, as results obtained in these 3 genotypes were not different.

### Intravital imaging of blood flow.

To analyze blood flow dynamics in vivo in response to pericyte stimulation in the ME, mouse ME was exposed by surgery as described (7, 45). Briefly, animals were anesthetized by injection of ketamine/xylazine (0.1/0.02 mg/g), then placed on a heating pad. Heart rate was monitored continuously and respiration was controlled by tracheotomy. The ventral side of the brain was exposed by drilling a hole in the palate bone and superfused continuously with NaCl 0.9% solution. Vessels were labeled by i.v. injection of fluorescent dextran molecules (labeled with D2, 150 kDa, 25 mg/mL in NaCl 0.9%, 100 μL/20 g body weight), and fluorescence was captured using an epi-fluorescence microscope fitted with a fast sCmos camera (ORCA Flash4.0, Hamamatsu) and a long working distance objective (2 cm, Mitutuyo, M Plan Apo ×20, NA 0.4) (45). Simultaneously, pericyte electrical activity was controlled in vivo using computer-controlled light flashes (50 ms, 1 Hz, 473 nm). Light was delivered to the tissue using optic fiber of 200 μm diameter placed at a maximum distance of 0.5 mm.

### Image data analysis.

Single *Z*-plane 1-minute movies were acquired every 5 minutes during 60 minutes. Blood flow in basal conditions was measured for 10 minutes before switching on the laser. A movie was then acquired every 5 minutes during 40 minutes of continuous laser stimulation (50 ms flashes at a frequency of 1 Hz). Acquisition rate was set to 150 frame/s. Movies were stabilized using ImageJ (NIH). Measurements were performed in at least 3 independent experiments per condition. The displacement of RBC shadows was visualized by contrast and quantified using previously described image analysis methods to calculate RBC velocity (45). RBC velocities were measured in 5–10 vessels per movie in at least 3 different mice per condition. Changes in vessel diameter induced by optogenetic stimulation of pericytes were measured using ImageJ (line scan function). Five to 10 vessels were measured prior to laser stimulation and 60 seconds after switching on the continuous laser stimulation in at least 3 independent movies from 3 different mice per condition.

### Fabrication of optic fibers.

Optic fiber implants were built by gluing a multimodal optic fiber (Thorlabs) with 200 μm core into a ceramic ferrule using high-temperature curing epoxy (Precision Fiber Products) as previously described (46). Fibers were polished and tested before implantation, to ensure optimal light transmission.

### Implantation of optic fibers.

Mice were anesthetized with 10 μL/g body weight of a mix of 1% ketamine and 0.1% xylazine in 0.9% NaCl. The head of the mouse was then blocked onto a stereotaxic frame and sterilized with ethanol and 10% betadine. Optical gel (Lacrigel, Europhta) was used to prevent dehydration of the eyes. A sagittal incision was made through the skin to expose the cranium, and then the head was positioned so that the sagittal suture lay horizontally in the rostro-caudal axis. After performing a small craniotomy using a dentist’s drill at the implantation site, 3 jeweler’s screws (Plastics One) were fixed to the skull to improve stability of the head cap. The optic fiber was then lowered through the brain to reach the stereotaxic coordinates (relative to bregma) –1.3 mm rostro-caudal, 0 mm medio-lateral, 5.7 mm ventral and finally fixed to the skull with Dentalon dental acrylic (Phymep). The mouse was left to recover in its cage for at least a week before initiating the experiments. Postoperatory analgesia was provided as an i.m. injection of ketoprofen. After surgery, animals were housed separately and checked daily. To minimize intra-animal variability, repeated analyses were made on the same mice, leaving at least 1 week between each experiment.

### In vivo peptide treatment.

Single-housed mice were attached to the stimulating laser through an optic fiber attached to a swivel and left to acclimate 3–4 hours. Ghrelin (rat, mouse, Phoenix Pharmaceuticals) was then injected i.p. (1 μg/g mouse), and food intake was measured over the course of 1 hour. Number of feeding episodes (manually counted as number of bites taken from food pellets) and total amount of food consumed (measured by weighing food pellets before ghrelin injection and 1 hour after) were monitored.

To assess in vivo diffusion of fluorescent molecules, either fixable 10 kDa dextran-FITC (MilliporeSigma) or bioactive fluorescent ghrelin derivative (coupled to a red fluorescent probe) developed by Cisbio Bioassays in collaboration with the Institut des Biomolécules Max Mousseron, Montpellier, France (7, 47), was used. Fixable 10 kDa dextran-FITC (3 mg/animal) or red ghrelin (25 nmoles/animal) was injected i.v. into the tail vein of either control (WT, NG2-Cre, or ROSA26-ChR2-Tomato mice) or NG2-ChR2 mice that were implanted with optic fibers at least 1 week before, fasted for 24 hours, left to habituate with optic fibers 3–4 hours, and subjected to 30 minutes of continuous laser stimulation (50 ms, 1 Hz, 200 μm fiber, 473 nm). Laser stimulation was continued and animals were sacrificed 5–10 minutes after ghrelin injection. Images were acquired using the same parameters between the different groups, and brightness and contrast were adjusted to the same levels between all images. Areas of diffusion of fixable 10 kDa dextran-FITC, corresponding to areas in which green fluorescent signal was detectable, were measured using ImageJ in image projections of the same thickness, or numbers of ghrelin-positive cells were counted, respectively.

To test for c-Fos upregulation in vivo, 0.9 % NaCl or commercial rat ghrelin (25 nmol/mouse, rat, mouse; Phoenix Pharmaceuticals) was injected in either control (WT, NG2-Cre, or ROSA26-ChR2-Tomato mice) or NG2-ChR2 mice that were implanted with optic fibers at least 1 week before, left to habituate with optic fibers 3–4 hours, and subjected to 30 minutes of continuous laser stimulation (50 ms, 1 Hz, 200 μm fiber, 473 nm). Laser stimulation was continued and animals were sacrificed 2 hours minutes after ghrelin injection. c-Fos–positive nuclei were counted using ImageJ in 20 μm projection of ME/ARH slices.

### Confocal imaging.

Terminally anesthetized mice were perfused via the heart with 10 mL of PBS followed by 30 mL of 4% paraformaldehyde solution. In some experiments, vessels were labeled using FITC-labeled lectin (400 μg per mouse, Vector Laboratories) diluted in the perfusate (PBS). Brains were collected and prepared for confocal imaging (7). Antibodies used were anti–PDGFR-β (1:200, R&D Systems, Bio-Techne; reference AF1042), anti–α-SMA (1:200, Abcam, reference ab5694), and anti–c-Fos (1:1,000, Santa Cruz Biotechnology, reference sc-166940). Nuclei were labeled using DAPI (MilliporeSigma). One to 4 slices were randomly selected from more than 3 animals/group. Images were acquired using a Zeiss LSM 780 confocal microscope and analyzed using Imaris (Bitplane) and ImageJ (NIH).

### Single-cell RNA-Seq data analysis.

Data from Campbell et al. 2017 (23) were imported in R 4.1.3 from the scRNAseq Bioconductor package version 2.7.2, then converted to a Seurat object, for plotting using the Seurat package, version 4.1.1. The cell labels were imported from the metadata and renamed according to what was reported in the original article.

### Statistics.

Values are represented as mean ± SEM. Statistical tests were performed using GraphPad Prism. Normality was tested using D’Agostino-Pearson test, and comparisons were made using either unpaired 2-tailed Student’s *t* test, or 2-tailed Mann-Whitney *U* test, as appropriate. Multiple comparisons were made using 1-way or 2-way ANOVA followed by Bonferroni’s post hoc test. *P* values were considered significant at *P* < 0.05. Feeding data analysis was performed with R; mixed-effect models were generated using either food intake or delay as outcomes, the presence of implant and laser as fixed factors, and the animal as a random factor.

### Study approval.

Animal studies were conducted according to the European guidelines for animal welfare and were approved by the Institutional Animal Care and Use Committee (CEEA-LR-12167, Région Languedoc-Roussillon, Montpellier, France).

## Author contributions

NR and MS designed experiments; NR, CL, PC, AG, TF, and MS performed experiments; NR, DJH, and MS analyzed data; and NR, DJH, PM, and MS wrote the manuscript.

## Supplementary Material

Supplemental data

Supplemental video 1

Supplemental video 2

## Version 1 12/27/2022

In-Press Preview

## Version 2 02/08/2023

Electronic publication
