# Supplementary material for: Median eminence blood flow influences food intake by regulating ghrelin access to the metabolic brain
Source: JCI Insight. 2023 Feb 8;8(3):e165763. doi: 10.1172/jci.insight.165763 (PMC9977422; doi:10.1172/jci.insight.165763)
Supplement: Supplemental data [file jciinsight-8-165763-s070.pdf]

SUPPLEMENTAL DATA

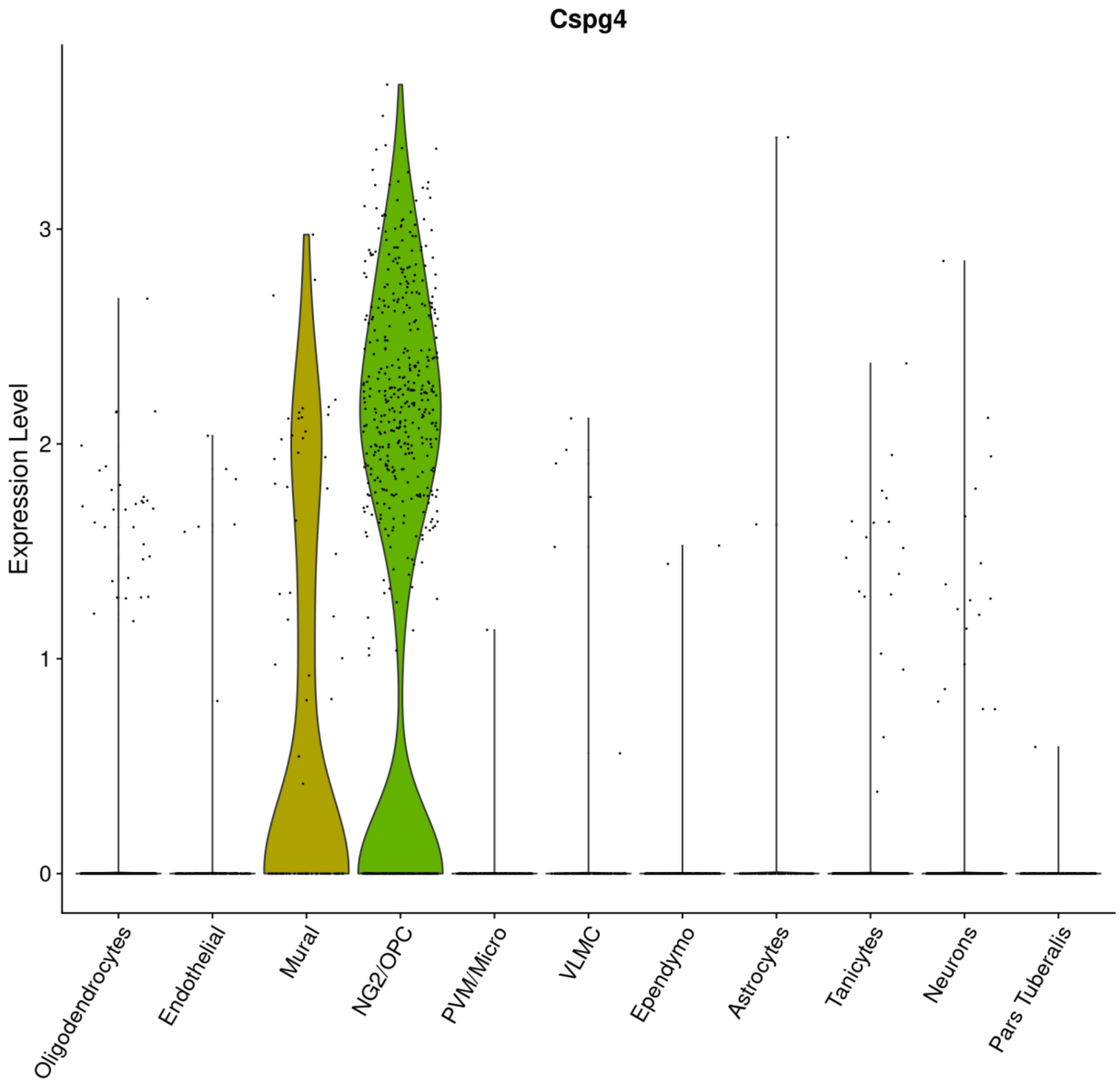

Supplemental Figure 1. Boxplot of normalized expression level for *Cspg4* (gene coding for NG2) in the ARH/ME cell populations identified in Campbell et al., 2017. *Cspg4* expression is confined to the NG2/OPC and mural cells clusters, while it is essentially absent from other cell populations, including endothelial cells (n = 26774 cells), (OPC: oligodendrocyte precursor cell, PVM: peripheral vascular macrophage, VLMC: vascular and leptomeningeal cell, ependymo: ependymocyte).

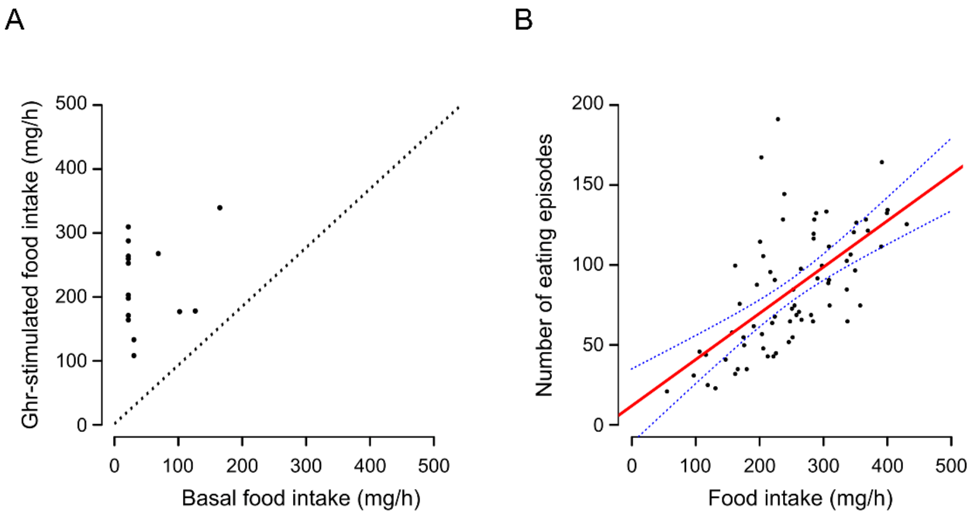

Supplemental Figure 2. Ghrelin-stimulated hourly food intake is linearly correlated with number of eating episodes. A) Relation between basal (non-stimulated) food intake and ghrelin stimulated food intake. B) Hourly food intake is linearly correlated to the number of eating episodes (red line shows linear regression, adjusted $R^2 = 0.38$; blue lines show 95% confidence intervals) (n = 57 experiments from 19 different mice).

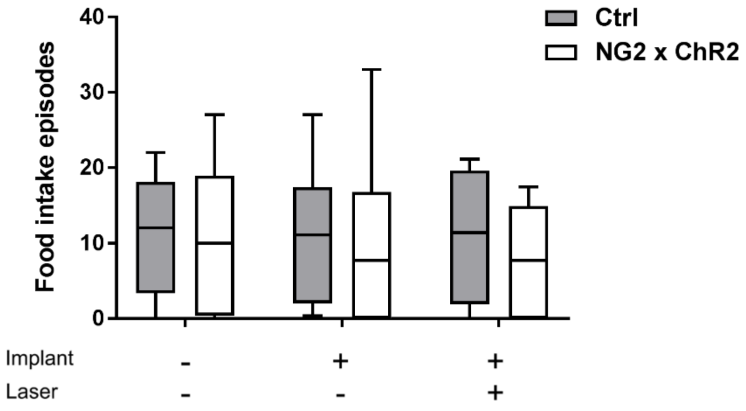

Supplemental Figure 3. Optogenetic stimulation of NG2-positive cells at the ME does not affect basal feeding behavior. Total number of food intake episodes over the course of 1 h (n = 4-11 mice/condition, mixed effect model). Boxes represent median with interquartile range, whiskers represent Min to Max range.

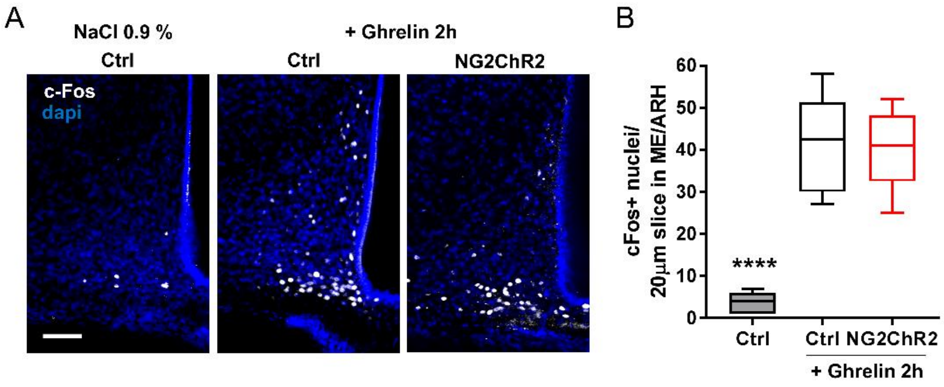

Supplemental Figure 4. Optogenetic stimulation of NG2-positive cells at the ME does not affect c-Fos activation in the ARH 2 h post-injection of ghrelin. A) Confocal images of ME/ARH region 2 h following injection of 0.9 % NaCl in a control mouse (left), ghrelin (25 nmoles/mouse) in a control mouse (middle) and in a NG2-ChR2-Tomato mouse (right) subjected to continuous laser stimulation at the level of the ME (473 nm, 50 ms, 1 Hz) (blue: dapi, white: c-Fos). Scale bar: 100 µm, 20 µm Z-projection. B) Quantification of the number of c-Fos positive nuclei in 20 µm projections of slices of the ME/ARH region (2-4 slices/mouse, n = 2 mice/condition). Ghrelin induces c-Fos activation in the ARH 2 h post-injection ($P<0.0001$, one-way ANOVA). Optogenetic stimulation does not affect number of c-Fos positive nuclei in the ME/ARH at 2 h post-injection of ghrelin in NG2-ChR2-Tomato mice (one-way ANOVA). Boxes represent median with interquartile range, whiskers represent Min to Max range.

SUPPLEMENTAL VIDEO LEGENDS

Video S1. Optogenetic stimulation in NG2-ChR2-tomato mice in vivo at the ME modifies blood flow. In vivo of blood flow in a control mouse (left) and a NG2-ChR2-Tomato mouse (right). Vessel parenchyma was labeled by i.v. injection of D2-labeled dextran (150 kDa). Laser flashes start at t = 6 s (473 nm, 50 ms, 1 Hz). Acquisition rate: 100 frames/s. Movie rate: 100 frames/s. Total elapsed time: 41 s. Image size: 442 × 192 µm, single Z-plane.

Video S2. Optogenetic stimulation in NG2-ChR2-tomato mice in vivo at the ME modifies vessel diameters. Example of in vivo contraction of vessels induced by optogenetic stimulation in a NG2-ChR2-Tomato mouse. Vessel parenchyma was labeled by i.v. injection of D2-labeled dextran (150 kDa). Laser flashes start at t = 10 s (473 nm, 50 ms, 1 Hz). Acquisition rate: 150 frames/s. Movie rate: 150 frames/s. Total elapsed time: 23 s. Arrow indicates vessel in which constriction is directly visible. Image size: 505 × 252 µm, single Z-plane.
